# I Won’t Forget to Do It If It’s Important: A Multinomial Processing Tree Analysis of Social Importance and Monetary Reward on Event-Based Prospective Memory

**DOI:** 10.5334/joc.367

**Published:** 2024-05-15

**Authors:** Geoffrey Blondelle, Véronique Quaglino, Yannick Gounden, Anaïs Dethoor, Harmony Duclos, Mathieu Hainselin

**Affiliations:** 1 CRP-CPO, UR UPJV 7273, Universitéde Picardie Jules Verne, Amiens, France; 2 INSPÉ de l’académie d’Amiens, Université de Picardie Jules Verne, Amiens, France

**Keywords:** prospective memory, motivation, mathematical modelling

## Abstract

While previous research has suggested that prospective memory may be enhanced by providing a social motive (i.e., social importance) or by promising a monetary reward for successful performance, to the best of our knowledge, the underlying mechanisms responsible for these effects are still largely unexplored. In a sample of 96 younger adults, this study investigated how social importance and promising a monetary reward influence the prospective component and the retrospective component of event-based prospective memory separately, with the application of a multinomial modeling approach. Results revealed enhanced prospective memory performance for all importance conditions compared to a standard condition. This improvement was characterized by an increased allocation of resource-demanding attentional processes in performing the prospective memory task at the expense of the ongoing task without an increase in the perceived importance of the prospective memory task. The model-based analyses showed that the beneficial effects of importance arise from an increased engagement of the prospective component, leaving the estimates for the retrospective component unaffected.

## Introduction

Event-based prospective memory (PM) refers to the ability to remember to perform an intended action (e.g., remembering to buy medication for your spouse on the way home) in response to a specific target event (e.g., seeing the pharmacy) while engaged in an ongoing activity (e.g., monitor the traffic while driving). Successful PM performance is thought to involve three distinct underlying processes: 1) the retrospective recall of *what* needs to be done (i.e., buy medication), 2) the recognition of the event or cues that indicate *when* the action needs to be performed (i.e., passing by the pharmacy) and 3) remembering *that* we are supposed to do something (i.e., I feel I have something to do). Together, *what* and the *when* constitute the retrospective component of PM, while the remembering *that* something needs to be done refers to the prospective component of PM. A key aspect of PM tasks is that they occur in the midst of some other ongoing activities that must be interrupted to perform the PM task. To simulate these real-life situations, laboratory PM tasks are embedded in an ongoing task (Einstein & McDaniel, 1990, see also Blondelle et al., 2020 for review on PM assessment instruments). When an event-based target cue occurs during the completion of the ongoing task (e.g., identifying the word *bald* when performing a lexical decision task), participants need to remember to perform an additional action (e.g., press the *P* key on the keyboard). Since the 2000s, researchers stressed the impact of the ongoing task on the PM task and highlighted the allocation of (limited) resource-demanding attentional processes away from the ongoing task in service of processing related to the PM task. They thus infer whether successful PM retrieval processes mainly involve a strategic allocation of attention (i.e., *enhanced ongoing task cost*; see Smith, 2003) or whether they occur spontaneously (i.e., without cost to the ongoing task; see McDaniel & Einstein, 2000; Scullin, McDaniel, & Einstein, 2010). Since then, there has been an increasing body of research that has examined the extent to which motivation – as measured through the importance attributed to the PM task – influences PM performance and the allocation of attentional resources.

Motivation is commonly distinguished between intrinsic motivation and extrinsic motivation (Ryan & Deci, 2000). Intrinsic motivation refers to an inherent interest in performing a given task, while extrinsic motivation refers to a means-end interest in performing a given task. Penningroth and Scott (2007) outlined the goal-based motivational-cognitive model of PM in which PM performance is influenced by goal representations. The model suggests that goal-related intentions will be more accessible in memory, benefit from an increase in the perceived importance of the PM tasks and thus improve PM performance either by both effortful attentional processes (i.e., strategic monitoring) or automatic processes (i.e., spontaneous retrieval). However, this model does not indicate whether strategic retrieval or spontaneous retrieval is required to contribute to successful PM performance.

### The effects of task importance on event-based PM

The importance of an intention is based on a complex interplay between the prior intention and other intentions, goals, desires, and the consequences of failure or the benefits of success for oneself or others associated with that intention (Kvavilashvili & Ellis, 1996). For the purpose of the study, we confine the scope on the role of providing a social motive and a monetary reward and their effects on both the prospective memory task and the ongoing task (for reviews, see Peter & Kliegel, 2018; Walter & Meier, 2014).

### The effects of promising a monetary reward on event-based PM

The effects of promising a monetary reward on event-based PM are mixed with some studies finding improved performance (Horn & Freund, 2021; Krishnan & Shapiro, 1999; McCauley et al., 2009; Somerville et al., 1983; Walter & Meier, 2017) while other studies did not (Guajardo & Best, 2000; Kliegel & Martin, 2003). On the one hand, evidence of an increased engagement in effortful attentional processes to monitor for event-based PM cues under a monetary reward condition comes from a study in which only a self-reported measure was used (Krishnan & Shapiro, 1999). Another study has provided more compelling evidence on this issue by objectively measuring ongoing task costs using a lexical decision task (Walter & Meier, 2017). Compared to a standard condition without importance manipulation, the authors reported enhanced PM performance when a monetary reward was promised. This performance improvement did not come at costs on the ongoing task relative to the standard condition, suggesting that participants may have changed their resource allocation policies when they were informed to perform the PM task (as evidenced by slower ongoing task reaction times compared to a baseline condition without embedded PM target events) but not significantly when they were awarded a monetary reward.

### The effects of providing a social motive on event-based PM

Manipulating the social importance of the task by providing a social motive to participants also enhances event-based (Cook et al., 2015; Horn & Freund, 2021; Cicogna & Nigro, 1998; Walter & Meier, 2017) and activity-based (i.e., a variant of event-based task in which PM tasks that must be performed upon the completion of an activity) PM (Brandimonte et al., 2010; Brandimonte & Ferrante, 2015). Brandimonte et al. (2010) used an activity-based task and showed improved PM performance in the presence of a social motive (i.e., doing a favor for the experimenter) and this came at faster ongoing task performance, suggesting that PM retrieval for prosocial intentions relies on spontaneous retrieval processes. The authors also found that the introduction of a monetary reward for carrying out prosocial intentions decreased PM. In their follow-up study, Brandimonte and Ferrante (2015) reached a similar result using the same task as in their previous research. They showed that activity-based PM was specifically reduced when small rewards (1 euro) were used compared to higher rewards (20 euros) or a social importance condition alone (Experiment 1). Moreover, PM performance was lower when a non-material reward, which consisted of disclosing the altruistic nature of participants’ behavior, was provided (Experiment 2). While the ongoing task was performed faster in the social importance condition (Experiment 1), it was performed slower when a non-material reward was introduced (Experiment 2). The authors hypothesized that the beneficial effect of social importance could be modulated unconsciously when the reward amount is manipulated or consciously when a non-material reward is introduced.

Walter and Meier (2017) examined the effects of social importance on event-based PM performance and ongoing task costs and showed enhanced PM performance in all importance manipulation conditions (i.e., social importance, monetary reward and both) compared to the standard condition. There were no additional ongoing task costs in the social importance condition and the monetary reward condition alone compared to the standard condition, suggesting that only the presence of PM target events changed participants’ allocation of attention. Nevertheless, in contrast to Brandimonte studies, their results revealed greater ongoing task costs in the combined condition relative to the standard condition. Although this effect appears to stem from the distinct demands of the event-based task (compared to activity-based tasks), the authors suggested it could result from conflicting motives of which participants were aware, due to the tangible nature of the proposed rewards.

A recent study investigated the combined effect of social importance (possibility of donation) and monetary reward with contingent gains and losses based on performance in both young and older participants using an event-based non-focal PM task to (Horn & Freund, 2021; Experiment 2). In the gain-frame condition, participants were told they could earn an extra 2.5 USD by responding accurately to a certain percentage of PM targets, gaining 25 cents for each correct response. In the loss-frame condition, participants were informed they initially received 2.5 USD but would incur deductions of 25 cents for every missed PM target. The authors also measured the perceived importance of the PM task and demonstrated improved PM performance for individuals who rated the task as more important. Additionally, their findings indicated that loss-frame condition and task importance contributed to better PM performance (with this effect being even more pronounced among older participants). Typically, this value-related improvement in PM performance did not came at increased costs on the ongoing task (see also Cook et al., 2015 for similar results). The authors used cognitive modeling techniques (see the section below for further details) to determine whether the source of the beneficial effects associated with the manipulation of importance resulted from increased engagement of preparatory attentional processes (i.e., the prospective component of PM) and/or enhanced discrimination of PM targets from non-target events (i.e., the retrospective component of PM). Their findings demonstrated that manipulating the importance of the PM task led to an improvement of the prospective component in young participants without the retrospective component being affected by the different experimental manipulation in this group. These findings seem to support the idea that social importance and promising a monetary reward enhance event-based PM performance without any additional cost to the ongoing task compared to a standard condition without any manipulation of importance. However, it remains unclear how these types of motivational incentives, taken in isolation, influence the PM retrieval its components, notably in tasks mediated by strategic monitoring (i.e., non-focal PM task).

### The multinomial model of event-based PM

Based on the PAM theory, Smith and Bayen (2004) developed a multinomial processing tree model (MPT) of event-based PM that offers the potential to provide parameter estimates engaging both the prospective component (i.e., the preparatory attentional processes) and the retrospective component (i.e., the ability to discriminate the PM targets from the non-targets events) to investigate how some variables affect each of them.

**Figure 1:**
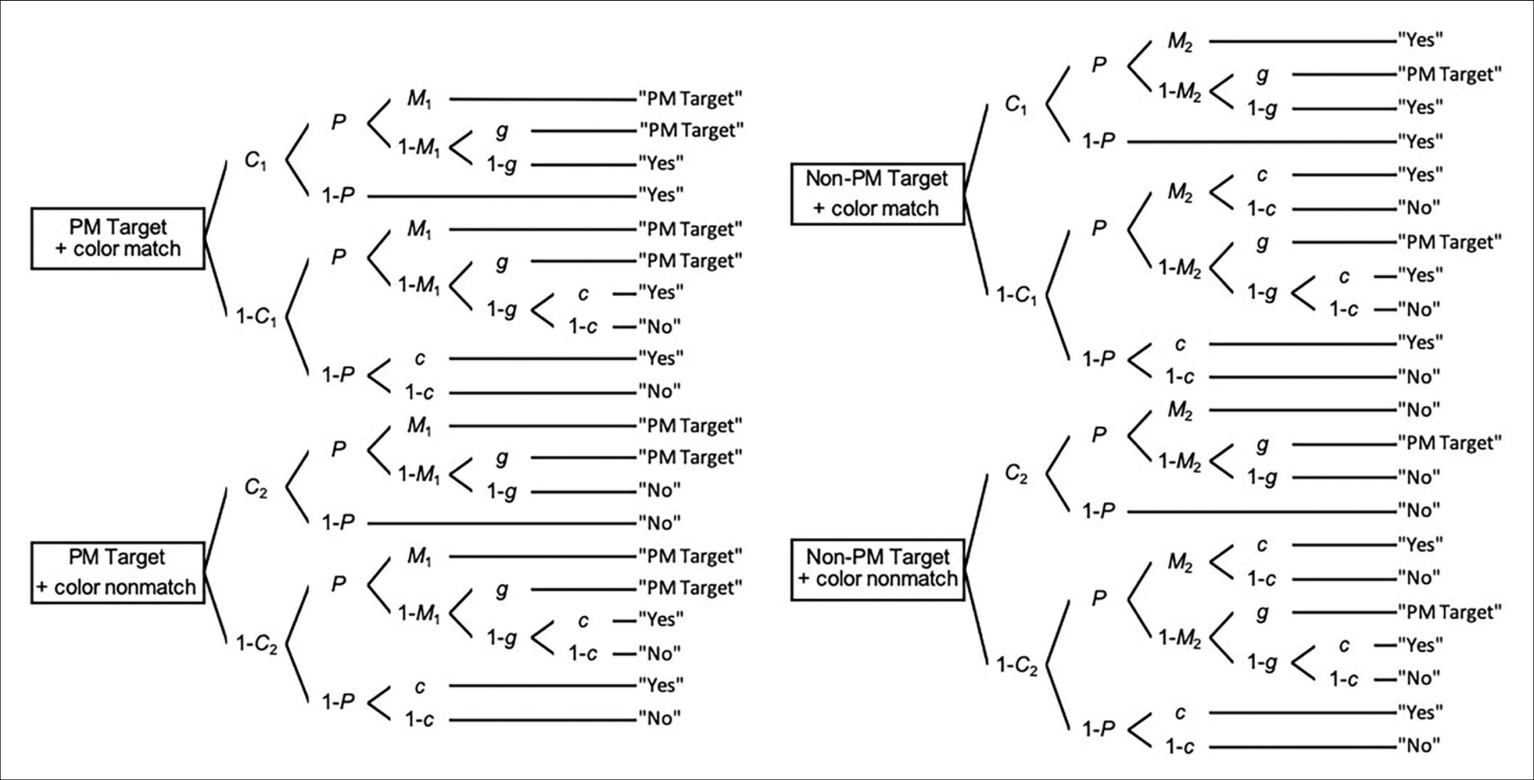
MPT model of event-based PM. *Notes*. PM = Prospective Memory. In the color-matching task, there were four different trial types: 1) PM targets with match trials, 2) PM targets with non-match trials, 3) non-PM targets with match trials and 4) non-PM targets with non-match trials. The top left portion of the tree in Figure 1 refers to PM targets that occur in color-match trials. In the upper half of the tree, *C*_1_ refers to the probability that participants detect the color of the word as a match. *P* refers to the probability that participants engage the prospective component whereas 1 – *P* refers to the probability of failing to engage the prospective component. *M*_1_ refers to the probability of engaging a successful recognition of the PM target, resulting in a ‘PM’ response. In the case where participants do not recognize the target (i.e., 1 – *M*_1_), they can either guess that the word is a target (*g*), resulting in a ‘PM’ response or not (1 – *g*), thereby resulting in a ‘match’ response. In the case where participants successfully detect that the color of the word match but failed in engaging the prospective component (i.e., 1 – *P*), they cannot produce a ‘PM’ response, resulting in a ‘match’ response. The lower half of the tree refers to the case in which participants fail to detect that the color of the word matches (i.e., 1 – *C*_1_). In this condition, participants may successfully engage the prospective component (*P*) and recognition of the target (i.e., *M*_1_), resulting in a ‘PM response’. Once again, if the participants do not recognize the target (i.e., 1 – *M*_1_), they can either guess that the word is a target (*g*), resulting in a ‘PM’ response. In the case where participants do not guess that the word is a target (1 – *g*), they may either guess with probability c that the color of the word matches, resulting in a ‘match’ response or guess with the probability 1– *c* that the color of the word does not match, therefore resulting in a ‘non-match’ response. In the case where participants fail to engage the prospective component (i.e., 1 – *P*), they may either guess (*c*) that the word matches, resulting in a ‘match’ response, or they may guess that the color of the word does not matches, therefore resulting in a ‘non-match’ response. Adapted from ‘A multinomial model of event-based prospective memory’ by R. E. Smith and U. J. Bayen, 2004, *Journal of Experimental Psychology: Learning, Memory and Cognition*, 30, p. 758.

The MPT model of event-based PM includes seven parameters: *C*_1_, *C*_2_, *P*, *M*_1_, *M*_2_, *g* and *c* (see Figure 1 for an overview of the MPT model used in the current study). Smith and Bayen (2004) have nicely demonstrated in their initial study that the seven-parameter version of their model was not identifiable. This requires the application of theoretically motivated restrictions to the model. The idea behind this is that participants who perform a task usually calibrate their guesses to the perceived ratio of targets to distractors, also defined as probability matching (Spaniol & Bayen, 2002). In line with this assumption, we would have to apply certain constraints to the model parameters in order to reach a globally identifiable model. This involves either setting predetermined values to certain parameters (Erdfelder & Buchner, 1998), or may also involve setting equal constraints between at least two parameters (Batchelder & Riefer, 1990). Thus, we have set the value of the parameter *c* according to the proportion of match trials and the value of the parameter *g* according to the proportion of targets (i.e., *c* = .50 and *g* = .08 in the current experiment). The application of these constraints has led to a model which is globally identifiable and testable through four parameters: *C*_1_, *C*_2_, *P* and *M* (see Smith & Bayen, 2004 for the mathematical proof of identifiability).

## The current investigation

The goal of the current study was to further investigate the effects of providing social motives and a monetary reward on event-based PM performance by studying them separately and their influence on the prospective and the retrospective component of PM. First, we tested whether this performance improvement (typically associated with no additional costs on the ongoing task) compared to a standard condition without any manipulation of importance could also apply to tasks that are mediated by strategic monitoring such as non-focal event-based tasks. In fact, the goal-based motivational cognitive model (Penningroth & Scott, 2007) predicts that goal-related intentions increase PM retrieval through more or less effortful monitoring of PM targets. However, it does not specify how motivational incentives influence the recall of PM target in tasks mediated by strategic monitoring, as this is the case for non-focal PM tasks (Smith & Hunt, 2014). Based on the PAM theory (Smith, 2003), we predicted that manipulating the importance of the task would increase PM performance due to a change in resource allocation policies, and thus increased strategic monitoring. Second, we applied the MPT of event-based PM (Smith & Bayen, 2004) to examine the effects of social importance and promising a monetary reward on both the prospective component and the retrospective component of event-based PM. If improved PM performance is expected to arise from a change in resource allocation policies toward the PM task, this effect should be observed through an increased engagement of the prospective component. We also predicted that the retrospective component would not be affected by the different importance manipulations because we controlled the target encoding to ensure consistent encoding across all conditions.

## Material and method

### Participants and design

The initial sample was composed of 96 young participants aged between 18 and 28 years. They were students from the University of Picardie Jules Verne (Amiens, France) and community-dwelling volunteers recruited by flyers. Participants gave their written consent and were informed that they could withdraw from the study at any time. They did not receive any financial compensation for their involvement in this study. All participants were (1) native French speakers (2) had normal or corrected vision with no visual color impairment (Ishihara & Force, 1943) and (3) no personal history of psychiatric illness and neurological disease. None of the participants presented signs of global cognitive deterioration, as determined by the Mini Mental State Examination (i.e., scores > 27/30; Folstein, Folstein, & McHugh, 1975). One participant was excluded and replaced due to the presence of color blindness.

The participants were randomly assigned to one of the four conditions: 24 participants received instructions that emphasized social importance, 24 received instructions that promised a monetary reward and 24 were given both the previous instructions simultaneously. Another group of 24 participants did not receive any additional instructions that emphasized the importance of the task after presenting the standard instructions of the PM task.

The final sample was then composed of 96 participants (*M* = 21.40 years, *SD* = 2.23; females = 66.67%) who did not differ across the four importance groups in gender distribution χ^2^(3) = 2.60, *p* = .458, age, *F*(3, 92) = 0.66, *p* = .580, η^2^*_p_* = .02, years of education, *F*(3, 92) = 0.71, *p* = .547, η^2^*_p_* = .02, and scores on the Mini Mental State Examination, *F*(3, 92) = 0.92, *p* = .434, η^2^*_p_* = .03.

### PM task and materials

**Figure 2:**
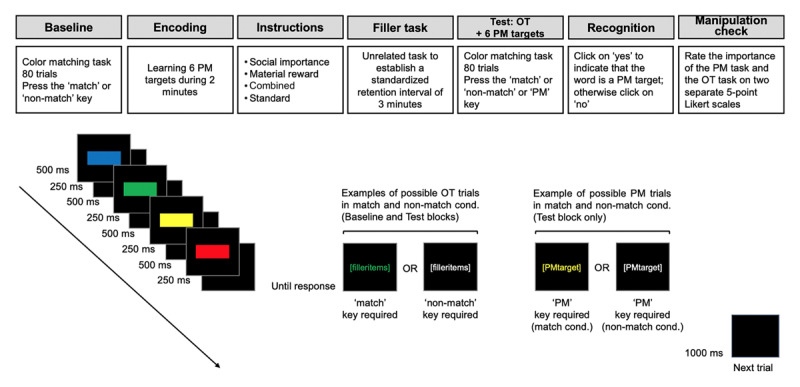
Illustration of the general procedure used in the current experiment and example of a trial with the possible responses. *Notes*. During the course of an ongoing task (OT) trial, the appearance of a green filler item results in a *match* response to indicate that the color of the item matches the color of the second rectangle presented just before. Conversely, the appearance of a white filler item results in a *non-match* response because the color does not match any of the rectangles presented. For the prospective memory (PM) task, participants are required to ignore the responses associated with the color match so that the appearance of PM target results in a *PM* response.

We embedded several non-focal target words in an ongoing color-matching task, a classic PM paradigm (Smith & Bayen, 2004, 2006; Smith & Loft, 2014). This task is supposed to be cognitively demanding and compatible with the application of MPT techniques. In the ongoing task (see Figure 2), participants saw a series of four colored rectangles presented successively on the screen, followed by a colored word. In the ongoing color-matching trials, participants were asked to indicate at the end of each trial whether the color of a word matched (or not) with one of the four colored rectangles that were presented just before. For the PM task, participants were asked to press a designated key whenever a specific target word appeared (e.g., the word *bald*) instead of generating an ongoing task response.

We selected a large set of 160 French medium-frequency words (*M* = 365.81, *SD* = 16.21) from the LEXIQUE 3.82 database (New et al., 2004). Each word was composed of between 6 and 8 letters in length to ensure that words were efficiently processed (for more details, see Schuster et al., 2016). From this set, we randomly selected 6 PM target words matched for frequency and word length. We used multiple PM targets because it increased both the ability to detect potential effects and the reliability of the prospective tasks (Kelemen et al., 2006; Maylor, 1996). The use of six PM targets is also consistent with previous PM research (cf. Lee et al., 2017; Smith & Bayen, 2004, 2006; Smith et al., 2014). The remaining 154 words served as filler items for the ongoing task. We created a baseline block composed of 80 filler items and a test block composed of 74 filler items in which the 6 PM targets were embedded. Paired Student’s *t*-test revealed no significant difference in word frequency between the two blocks (*t* < 1). The PM target words included in the test block appeared randomly on trials 10, 23, 36, 49, 62, and 75 for a total of 80 trials per block. The order of presentation of the filler items was randomized within the baseline block and the test block, as was the presentation of the PM targets in the test block and during the encoding phase. The block administration order was counterbalanced within each instruction group for controlling for practice and fatigue effects when interpreting costs in a within-subject design. Thus, half of the participants performed the baseline block first and then moved onward to the test block, whereas the other half of the participants performed the test block first and then moved onward to the baseline block.

Five following colors were used for both the rectangles and the words of the color-matching task: red, yellow, green, blue and white. In the ongoing color-matching task, the colored rectangles (size: 3.81 cm × 3.30 cm) appeared in the center of a 15-inch black screen and the colored words appeared in lowercase letters in 18-point font size. All programming was done with E-Prime 3.0.3.31 software (Schneider et al., 2002) and a Chronos 5-button response box (which provided the accuracy of a few milliseconds on all machines) was used to record both reaction times (RTs) and accuracy.

### Procedure

The general procedure of the experiment is illustrated in Figure 2. After signing the consent forms, participants completed the demographic questionnaire and were then proposed the Mini Mental State Examination and the Ishihara test. The color-matching task was administered in an individual session, with each participant seated in front of a computer and a Chronos response box. The instructions of the color-matching task were displayed on the screen and they emphasized both speed and accuracy. In each color-matching task trial, participants saw four colored rectangles consecutively displayed in the center of a black screen followed by a colored word. For half of the trials of the color-matching task, the color of the word matched with one of the rectangles presented just before (match trials) and in the other half, the color did not match with any of the previously presented rectangles (non-match trials). There was the same proportion of match and non-match trials in each block (i.e., practice, baseline and test) of the color-matching task. For each trial, the color of the rectangles was randomized. Each colored rectangle was presented during 500 ms and followed by a 250 ms blank screen. After the fourth rectangle and the fourth interstimulus interval of 250 ms, a colored word appeared in the center of the screen and participants were requested to judge whether or not the color of the word matched with one of the previous rectangles by pressing the ‘match’ key or the ‘non-match’ key on the Chronos response box. Participants entered their responses using the leftmost and the rightmost of five adjacent keys on the Chronos response box. Both the leftmost and the rightmost keys of the Chronos response box were activated and designated as ‘yes’ to indicate a *match* trial and ‘no’ keys to indicate a *non-match* trial. The colored word displayed on the screen stayed active until a response was entered and a blank screen was then displayed for 1000 ms, followed by the first rectangle of the next trial.

After reading the instructions of the color-matching task, participants completed a six-trial practice block (3 match trials and 3 non-match trials). If participants produced more than one incorrect answer out of the six trials performed in the practice block, the instructions for the ongoing task were displayed again on the screen and participants completed a second 6-trial practice block. No participant has completed the practice block more than three times. At the end of the practice block, we provided the opportunity for participants to ask questions to ensure that they understood the instructions of the ongoing color-matching task before proceeding to the baseline block or to the PM block instructions depending on the counterbalanced condition.

At the end of the baseline block, the participants were informed that they would have to learn the six target words. The six target words were displayed in the center of the screen for 2 minutes. After the encoding phase, the experimenter gave a small piece of paper to the participants and asked them to write the six target words that they had just learned before in any order they wished. The experimenter then collected the piece of paper on which the participants wrote the six target words before continuing the experiment. If a participant failed to recall all the six target words, the six target words were presented for 2 minutes for re-encoding and a second recall were carried out. No participant performed more than three recall trials to retrieve the six target words.

The participants were then informed that they would have to perform an additional task in the following block of the ongoing task. At this time, the central button of the response box was activated (in addition to the leftmost and rightmost keys) and was designated as the ‘PM’ key. The participants then received the instructions of the prospective task. In the standard condition, the following instructions were displayed on the screen (translation from French): ‘*When the color-matching task will resume* [not announced in the counterbalanced condition] *please additionally remember to press the yellow key on the response box instead of the green key or the red key when you see one of the six words learned before*. *Please show me the key associated with the “PM” response to the response box*.’ In the social condition, the additional sentence was given ‘*If you remember to press the yellow key every time you see one of the six words*, *this will generate important information for all patients that will be included later in this study’*. In the monetary reward condition, the following additional sentence ‘*If you remember to press the yellow key every time you see one of the six words, you will earn 10 euros*’ was added. In the combined condition, both additional sentences ‘*If you remember to press the yellow key every time you see one of the six words, you will earn 10 euros and this will generate important information for all patients that will be included in this study*’ were added. It was clearly stated that the reward would be provided only to those participants who have correctly pressed the ‘PM’ key when they noticed each of the 6 words during the test block. The experimenter then asked participants to explain in their own words the instructions for both the ongoing task and the prospective task to ensure that they had understood them.

After receiving the instructions of the prospective task, participants were requested to complete a letter-comparison task (Salthouse & Babcock, 1991) as filler task before starting the second block of the ongoing color-matching task. The letter-comparison task lasted about 3 minutes and served as a filler task to avoid ceiling effect in PM performance (see Smith & Bayen, 2004, 2006 for a similar procedure). Indeed, introducing a delay between the encoding phase and the performance interval tends to reduce PM performance (Raskin et al., 2012; Raskin & Sohlberg, 2009). In the letter-comparison task, participants saw two sets of letters displayed in the center of the screen and they had to indicate whether or not the two sets were identical. Participants performed three blocks of the letter comparison task with different levels of complexity (i.e., with series composed of 3, 6 and 9 letters). After 60 s, participants automatically switched to the next block to standardize the delay between the encoding phase and the test phase.

At the end of the letter-comparison task, participants began the test block on the ongoing color-matching task in which the six target words were embedded. The instructions of the prospective task were not reminded before starting the test block. Following the test block, the participants of each PM instruction groups were asked to recall the key to be pressed when the target words appeared. No participant failed to remind that they had to press the ‘PM’ key on the response box as PM task.

After completion of the letter-comparison task, the participants were asked to perform an old-new recognition test in which they were requested to recognize successively the six target words included in the test block among 6 other filler words from the ongoing task trials (i.e., three filler words of each of the baseline block and test block). We have chosen to administer the recognition test at the end of the experiment in order to avoid overemphasizing the importance of the PM task. Finally, participants were asked to rate the importance of both the ongoing task and the PM task on a 5-point Likert scale (i.e., 1 = *not important at all* to 5 = *very important*).

### Data analysis

PM accuracy was calculated as the proportion of target events to which the participants made a correct “PM” response. Ongoing task RTs were examined on correct trials only. Moreover, the data from the PM target trials as well as the four following ongoing task trials were excluded from the analyses in order to control for potential confounding effects associated with switching costs (for a discussion on this issue, see Meier & Rey-Mermet, 2012). Therefore, it is assumed that the presence of a cost when performing the ongoing task cannot be due to the execution of the intention. Before analyses, ongoing task RTs data were trimmed by excluding RTs of less than 300 ms or 2 *SD* from individual participant means. This resulted in the exclusion of less than 2% of trials.

A series of one-way ANOVAs were conducted with jamovi (The jamovi project, 2020) to examine ongoing task performances, PM accuracy, participants’ post-test target recognition performance as well as importance ratings with the importance instruction groups (social importance, monetary reward, both and standard) as the between-subject factor. Note that participants’ performances on post-test assessment is often used in PM research through recognition or recall procedures to evaluate the retrospective component of PM. For these analyses, the rejection level for inferring statistical significance was set at *p* < .05.

To determine the fit of the multinomial model to the observed data, we first calculated participants’ frequency of responses for each item type (see Supplementary Table S1 for the response frequencies aggregated across participants and item types for each condition). Second, we calculated the parameter estimates *P* and *M* via maximum-likelihood estimation and goodness-of-fit index (*G*^2^), which is asymptotically χ^2^-distributed (Hu & Batchelder, 1994) for each of the four importance instruction conditions by using MultiTree software (Moshagen, 2010). Using G*Power3 (Faul, Erdfelder, Lang, & Buchner, 2007), with *N* = 1920 (24 participants × 80 trials) and four degrees of freedom, the alpha level of .05 produces the statistical power of .99 to detect even small deviations (effect size of *w* = .10; see Cohen, 1988). Corrections for multiple comparisons were made by adjusting the *p*-values according to the number of significance tests performed (i.e., *α*_corrected_ = .05/48 = .001).

## Results

### PM performance and post-test target recognition

**Figure 3:**
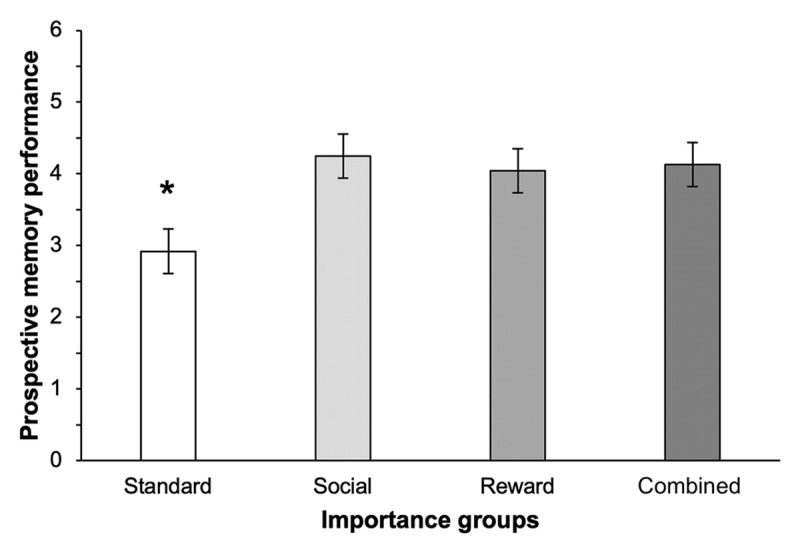
PM performance as a function of importance instructions. *Notes*. *Differs from all of the other importance conditions, *p* < .05. Error bars represent the standard error of the means.

The proportions of PM targets trials for which the participants correctly pressed the ‘PM’ key, presented in Figure 3, was significantly affected by importance instructions, *F*(3, 92) = 4.89, *p* = .003, η^2^*_p_* = .14. Tukey’s HSD revealed that, compared to the standard condition, participants’ PM performance were higher in the social importance condition, *t*(92) = 3.38, *p* = .006, the reward condition, *t*(92) = 2.85, *p* = .032, and in the combined condition, *t*(92) = 3.06, *p* = .017. However, participants’ PM performance in each of the three importance conditions did not differ from each other, *t*(92) < 1. There was no difference between the four conditions in the post-test target recognition test, *F*(3, 92) = 0.58, *p* = .631, η^2^*_p_* = .02.

### Ongoing task performances

#### Ongoing task accuracy

Ongoing task accuracy was examined with a 4 × 2 mixed ANOVA with the between-subject factor of importance instructions (standard, reward, social and the combined condition) and within-subject factor of color-matching trial type (match and non-match trials). There was no significant main effect of trial type*, F*(1, 92) = 0.66, *p* = .418, η^2^*_p_* = .01 and neither the main effect of instruction conditions, *F*(3, 92) = 1.10, *p* = .355, η^2^*_p_* = .04, nor the interaction between the two variables reached significance, *F*(3, 92) = 2.31, *p* = .082, η^2^*_p_* = .07. Due to the absence of significant differences in baseline accuracy, and in accordance with previous research (e.g., Heathcote, Loft, & Remington, 2015; Smith et al., 2014), we therefore subtracted participants’ mean accuracy in the baseline block from those in the test block. Results revealed that accuracy difference scores were neither affected by trial type, *F*(1, 92) = 1.30, *p* = .256, η^2^*_p_* = .01, nor by importance instructions, *F*(1, 92) = 0.33, *p* = .804, η^2^*_p_* = .01, and the two variables did not interact, *F*(3, 92) = 2.61, *p* = .056, η^2^*_p_* = .08, thereby suggesting that there was no cost on ongoing task accuracy when the importance of the PM task was manipulated.

#### Ongoing task response times

**Figure 4:**
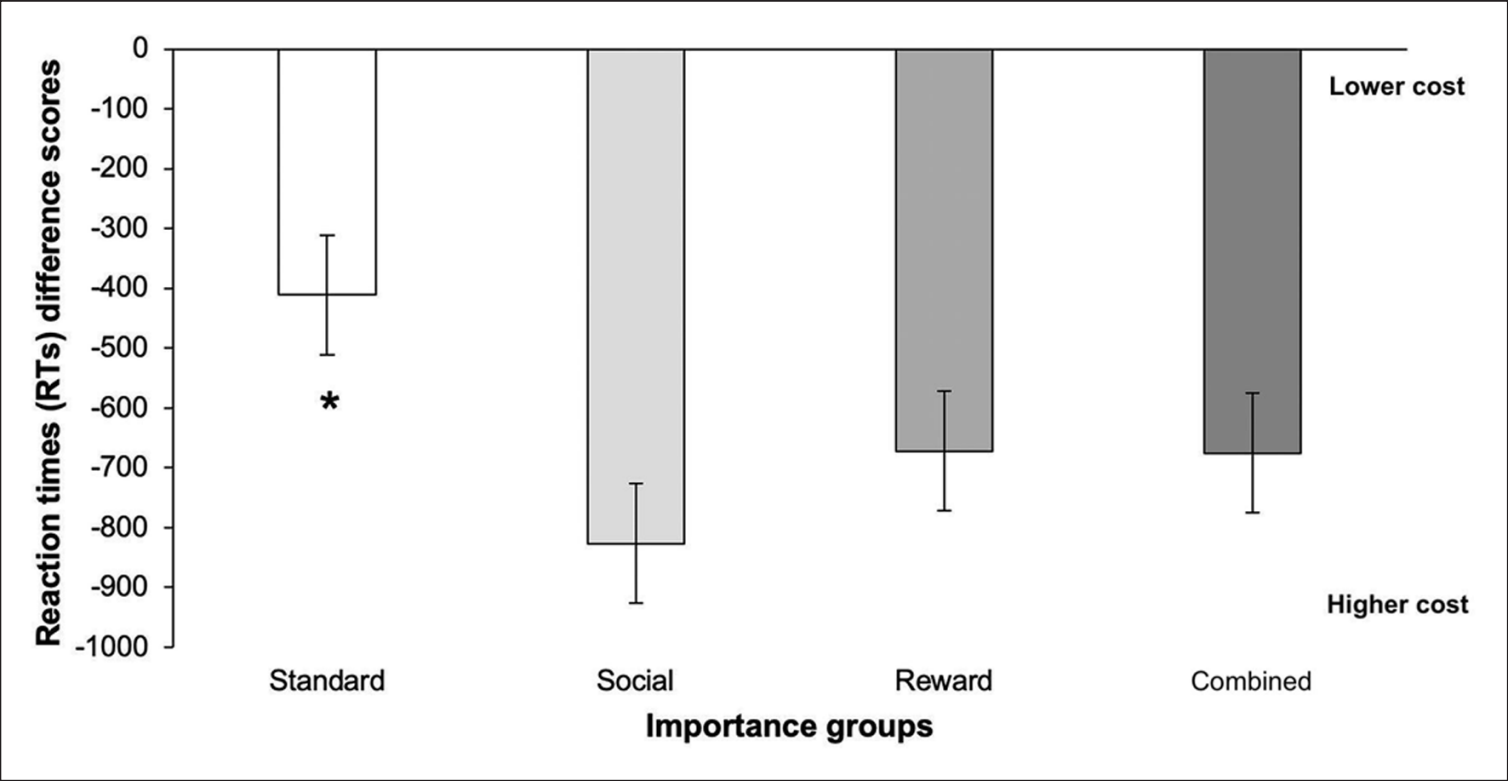
Reaction times difference scores (baseline block RTs – test block RTs) as a function of importance instructions. *Notes*. The lower reaction time difference, the higher the participants’ reaction time cost. *Differs from all of the other importance conditions, *p* < .05. Error bars represent the standard error of the means.

Ongoing task RTs were examined with a 4 × 2 mixed ANOVA with the between-subject factor of importance instructions (standard, reward, social and the combined condition) and within-subject factor of color-matching trial type (match and non-match trials). Baseline RTs were not different as a function of trial type, importance instructions and the two variables did not interact with each other (*F*s < 1, *p*s ≥ .49). Since there was no significant difference in baseline RTs, and as with baseline accuracy, we subtracted participants’ mean RTs in this block from those in the test block to compute RTs difference scores. RTs difference scores, displayed in Figure 4, were significantly affected by importance instructions, *F*(3, 92) = 9.82, *p* < .001, η^2^*_p_* = .24. Post hoc tests revealed that participants’ difference scores were higher (i.e., there was a lower ongoing task cost) in the standard condition compared to the social importance condition, *t*(92) = –5.33, *p* < .001, the reward condition*, t*(92) = –3.35, *p* = .006, and the combined condition, *t*(92) = –3.40, *p* = .006. However, there was no significant difference in participants’ difference scores between the three-importance conditions (Social vs. Reward: *t*(92) = –1.98, *p* = .202, Social vs. Combined: *t*(92) = 1.94, *p* = .219, and Reward vs. Combined: *t*(92) = 0.04, *p* > .999).

### Multinomial modeling results

We computed a common model for each importance instruction conditions in order to allow all parameters to vary between the four instruction conditions. Technically, each significance test was conducted by comparing a baseline model which imposes a restriction of equality between the instructions conditions on a given parameter (e.g., the parameter *P* and *M* in the standard importance condition was set to be equal between the four importance instruction conditions), yielding to a *G*^2^ statistic with a degree of freedom equal to 1.

**Figure 5:**
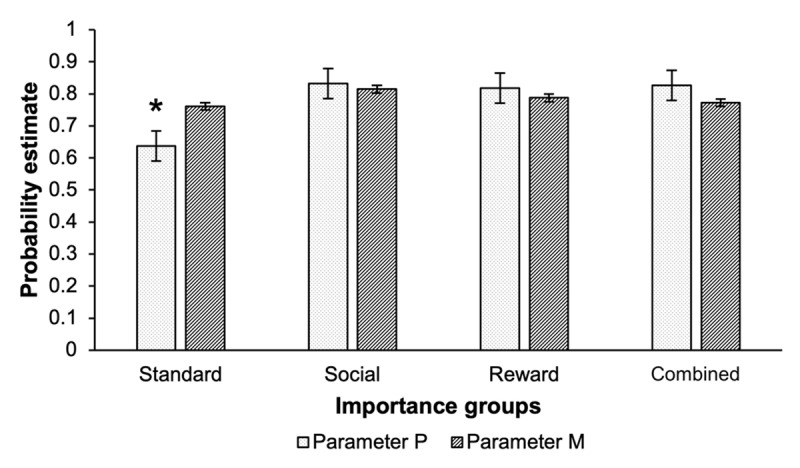
Parameter estimates of engaging the prospective component (P) and the retrospective component (M) of prospective memory as a function of importance instructions. *Notes*. *The parameter estimates for *P* differs from all of the other importance conditions*, p’* < .001; critical Δ*G*^2^ = 9.49. Error bars represent the standard error of the means.

The model provided a good fit of the data such that all values were smaller than the critical value of 9.49 in the standard condition Δ*G*^2^(4) = 2.44, *p* = .66, the social condition Δ*G*^2^(4) = 2.05, *p* = .73, the reward condition Δ*G*^2^(4) = 3.05, *p* = .55 and in the combined condition Δ*G*^2^(4) = 3.92, *p* = .42. The parameters *P* (probability of engaging preparatory attentional processes, referring to PM prospective component) and *M* (retrospective recognition processes, referring to PM retrospective component) are presented in Figure 5.

#### Prospective component of PM

The estimate for the parameter *P* was lower in the standard condition compared to the social importance condition Δ*G*^2^(1) = 21.73, *p* < .001, the reward condition, Δ*G*^2^(1) = 16.30, *p* < .001, and the combined condition, Δ*G*^2^(1) = 17.00, *p* < .001. However, the estimates for the parameter *P* were not statistically distinguishable between the three-importance groups (all Δ*G*^2^ values were ≤ .09 and *p*s ≥ .76).

#### Retrospective component of PM

The probability of engaging retrospective recognition processes of the PM targets did not differ between the four importance conditions (all Δ*G*^2^ values were ≤ 2.23 and *p*s ≥ .14).

### Manipulation check

The importance ratings of the PM task were *M* = 4.25 (*SD* = 0.68) for the standard condition group, *M* = 4.13 (*SD* = 0.90) for the social condition group, *M* = 4.04 (*SD* = 0.81) for the reward condition group and *M* = 4.13 (*SD* = 0.74) for the combined condition group. For the ongoing task, the importance ratings were *M* = 3.71 (*SD* = 1.16) for the standard condition group, *M* = 3.50 (*SD* = 1.32) for the social condition group, *M* = 4.04 (*SD* = 0.91) for the reward condition group and *M* = 3.54 (*SD* = 0.98) for the combined condition group. The importance ratings were examined with a 4 x 2 mixed ANOVA with the between-subject factor of importance instructions (standard, reward, social and the combined condition) and within-subject factor of task (PM task and the ongoing task). There was a main effect of task, *F*(1, 92) = 10.60, *p* = .002, η^2^*_p_* = .10, showing that the PM task was rated as more important compared to the ongoing task, *t*(92) = 3.25, *p* < .05, but neither the main effect of importance, *F*(3, 92) = 0.62, *p* = .605, η^2^*_p_* = .02, nor the interaction between the two variables reached significance, *F*(3, 92) = 1.19, *p* = .316, η^2^*_p_* = .04.

## Discussion

The current study is the first to demonstrate that social importance and/or promising a monetary reward improve non-focal event-based PM performance. This performance improvement came at greater costs on the ongoing task through increased engagement of the prospective component. Moreover, in all conditions, the PM task was perceived as more important relative to the ongoing task. More specifically, compared to the standard condition, PM performance improved across all the three-importance conditions (i.e., social importance, monetary rewards and combined condition). For the latter three conditions, this performance enhancement was also accompanied by greater costs on the ongoing task compared to the standard condition. MPT analyses yielded to the same pattern of results by revealing that the beneficial effects of task importance were explained by an increase in the prospective component of PM, which is thought to represent preparatory attentional processes, leaving the estimates for the retrospective component unaffected.

Our results revealed that event-based PM performance were higher in the three-importance conditions relative to the standard condition, extending the findings of a previous study using focal PM cues (Walter & Meier, 2017). Nevertheless, the improved PM performance in the social importance condition is not congruent with Altgassen et al.’s results (2010) who did not find such a benefit in young adults with time-based tasks (i.e., tasks that must be performed at a specific moment or after a given period of time). In their experiment, the nature of the social importance instructions (i.e., performing the PM task would be a favor for the experimenter to prepare another study) may have interfered with the obligation to complete the PM task and “reduced their subjective pressure to perform.” (p. 323). However, it appears that this phenomenon did not occur in our current study, and this may be due to the fact that the instructions did not involve a favor done to the experimenter himself. As mentioned above, the importance of an intention results from a complex interplay between the prior intention (i.e., completing the PM task) and the consequences of failures or the benefits of success for oneself or others associated with that intention (see Kvavilashvili & Ellis, 1996). We thought that younger participants in our study were more likely to perceive the PM task as socially relevant because the consequences arising from completing the PM task were associated toward more intrinsic values (i.e., performing the PM task well would benefit patients suffering from memory impairments). However, our data did not support this hypothesis because the improved PM performance found under the social importance condition was not accompanied by a greater perceived importance of the PM task compared to the standard condition, likely due to high levels of baseline intrinsic motivation of participants (see below). Future research should consider the impacts of the specific importance instructions or the operation of processes involved at each stage of intention processing.

Results showed greater ongoing task costs in all of the three importance conditions compared to costs that emerged in the standard condition. These results are in line with previous studies that have reported that manipulating the importance of non-focal PM tasks increased the allocation of conscious resources devoted to the PM task at the expense of the ongoing task performance (Horn & Freund, 2021; Kliegel et al., 2004; Smith & Bayen, 2004, Experiments 1 and 2; Smith & Hunt, 2014). However, Walter and Meier (2017) did not find greater ongoing task costs for both the social importance condition and the monetary reward condition relative to their standard condition. They concluded that providing a social motive as well as the prospect of a reward increases the likelihood of engaging spontaneous retrieval processes. While these conflicting results may be due to the non-focal nature of the event-based PM tasks, this could also be due to the way in which the target events were presented during encoding. As an example, we presented the non-focal tasks PM targets within a distinct encoding phase, but this was not the case in Walter and Meier’ study (2017). In their experiment, the participants were informed that they must press a designated key whenever a word denoting a musical instrument occurred during the task. Therefore, it is possible that the absence of such targets during encoding may have discouraged monitoring when they were embedded in the ongoing task in such a way that their appearance could not be anticipated, thereby reducing the retrospective memory load.

Our results also revealed that in the situation where the prospect of a monetary reward was added to a social importance condition (combined condition), ongoing task costs were comparable for both the social importance condition and the monetary reward condition but greater relative to the standard condition. This is in contrast with previous results where the prospect of a reward in a condition in which a social motive was provided to participants led to an additional cost when compared to the cost that emerged in the standard condition, whereas there were no differences between the social importance condition and the monetary reward condition alone compared to the standard condition (Walter & Meier, 2017). In our study, the absence of evidence for the crowding-out effect of intrinsic motivation can be attributed to the non-focal nature of the task, which may have been perceived as challenging by participants. Walter and Meier (2017) proposed a measure of task difficulty and observed that the PM task was perceived as more challenging than the ongoing task, without any significant effect related to importance being found. Unfortunately, we didn’t implement such a measure in the current experiment, but many participants mentioned during debriefing the high attentional demand nature of the ongoing task. Therefore, it is possible that the difficulty of the task by participants may have canceled out the crowding-out effect of intrinsic motivation in our study. We believe that it would be relevant to include this measure in future studies to shed light on this issue.

This study also extends the results of a previous study (Horn & Freund, 2021) by showing that PM improvement under a social importance and a material reward alone enhanced the prospective component of PM. In contrast to the standard condition, the model results revealed that the probability of engaging the prospective component was higher for all of the three-importance conditions, while the probability of engaging the retrospective component did not vary significantly across conditions. Extending the findings of previous studies that have emphasized the importance of non-focal event-based tasks (Smith & Bayen, 2004; Smith & Hunt, 2014), the ongoing task costs results are in line with the MPT model-based results. These ongoing task costs results are supported by the PAM theory (Smith, 2003), which showed that providing a social motive or promising a monetary reward enhances PM performance and this comes at the expense of the ongoing task performance. Importantly, greater costs on the ongoing task in all of the three-importance conditions were characterized by an increased engagement of the prospective component, which is thought to reflect the probability of engaging preparatory attentional processes. However, Horn and Freund (2021) did not find additional costs on the ongoing task in younger adults when external incentives were introduced. We believe that this divergence in results may be associated with differences between the paradigms used across studies. Indeed, in our study, the manipulation of importance was solely carried out through verbal instructions provided to participants, for which the incentives used were initially fixed, unlike the study conducted by Horn and Freund (2021) in which the reward received were performance contingent (in the form of gains and losses). These different manipulations can elicit different motivations and modulate ongoing task costs, either through increased strategic monitoring or through a reduction or elimination of monitoring. Taken together, these findings would suggest that the beneficial effects of motivation on PM retrieval may rely on strategic monitoring when incentives are fixed, whereas such monitoring may not be necessary (or expressed more subtly; cf., Scullin et al., 2013) in the presence of performance-contingent incentives to enable retrieval through increased accessibility of future intentions. Although speculative, these hypotheses require further examination and will help unveil a better understanding of how motivation can influence mechanisms of future intention retrieval. Finally, it is now clear that the greater ongoing task costs found in the importance conditions could not be due to rehearsal of the event-based PM targets during the task because the probability of engaging the retrospective component did not differ across conditions.

Although both the MPT model and the traditional behavioral measures were affected by the presence of a social motive and the prospect of a reward, this was not the case for the perceived importance of the task. Despite successful PM performance in the three-importance conditions, participants’ self-rated PM importance did not make it possible to distinguish between the standard condition and the importance conditions. This pattern of results was also found in a previous study (Walter & Meier, 2017) and suggests that these variables might reflect distinct motivational aspects. However, the measurement of perceived importance could offer insights into the level of participants’ intrinsic motivation, which appears relatively high in our study. This could be attributed to participants being informed that there was no compensation for their participation (see limitations section). Nonetheless, we believe it could be valuable to include measure of perceived importance, even if they don’t always align with observed behavioral motivational effects.

On a behavioral level, providing a social motive or promising a monetary reward tends to produce similar effects as the *implementation intention*, a self-regulation technique that benefits to goal achievement by forming intentions at the time of encoding in the form of *if*-*then* (Gollwitzer, 1999), such as “*if* I encounter the situation X, *then* I will perform the action Y”. Implementation intention is generally investigated by creating a link between the PM target event and the intended action. Previous research reported that implementation intention enhances event-based PM performance, and this came at greater ongoing task costs when non-focal cues were used (Meeks & Marsh, 2010, Experiments 1 and 2; Smith et al., 2014), but not for focal cues (McFarland & Glisky, 2012; 2011; Meeks & Marsh, 2010, Experiment 3). Our same pattern of results regarding non-focal event-based PM performance suggests that social importance, as well as promising a monetary reward, may have a similar impact as implementation intention on PM. Initial support for this idea stems from the fact that the standard PM instructions given to participants often take the form of implementation intention (e.g., press the Y key when a specific word occurs) in most paradigms. Although speculative, closer investigation examining the interplay between the different types of importance manipulation, implementation intention and cue focality will shed some light on this issue.

## Limitations of the current study

One of the limitations of this study is that participants did not receive any financial compensation for their participation in the study, either as part of their assignment to the reward condition or their assignment to the control condition. Regarding the first point, this may have introduced a participation bias, which may have resulted in only participants who were highly intrinsically motivated to engage in research without compensation to volunteering for our experiment. In support of this hypothesis, participants’ levels of intrinsic motivation, as assessed by perceived task importance in our experiment, did not differ between the importance conditions and the standard condition, with descriptively higher values in the latter condition. Regarding the second point, it is likely that the fact participants that did not actually receive 10 euros for perfect PM performance and they did not actually help the future patients included latter in the study may have led to some deception. Research in the field of experimental economics suggests that performance on attention-demanding tasks may be influenced by the real or hypothetical nature of the reward offered (see Camerer & Hogarth, 1999). Future studies investigating the links between PM and motivation should implement initial reward for all participants and vary this amount according to value-related performance, as has been done in some studies (e.g., Horn & Freund, 2021). Another limitation of this study was the use of the MPT model, which assumed that different groups of participants assigned to importance conditions are homogeneous and should have identical parameter values. Therefore, the results related to the MPT model in our study should be interpreted with caution.

## Conclusion

This study demonstrates that both social and monetary rewards enhance non-focal event-based PM without incurring additional costs on ongoing activities. Our findings indicate that these improvements are primarily attributable to an increased engagement of preparatory attentional processes, thereby bolstering the prospective component of PM. This suggests that appropriate incentives can induce changes in resource allocation policies without disrupting the retrospective component of PM, which facilitates a more efficient realization of intended actions. Future research should explore how motivational incentives influence prospective remembering in specific everyday life situations, such as in the context of medication adherence.

## Data Accessibility Statement

The data of the experiments provided in the manuscript have been uploaded to the Open Science Framework: https://osf.io/a28vn/?view_only=350ff0728e914e2c86d74973c0b237ca.

## Appendix

**Supplementary Table S1:** Response category frequencies as a function of trial type and importance manipulations.

IMPORTANCE INSTRUCTION CONDITION AND TRIAL TYPE	RESPONSE TYPE		

	MATCH	NONMATCH	PROSPECTIVE MEMORY

**Standard**			

Target, match	44	7	52

Target, nonmatch	4	45	47

Nontarget, match	692	146	13

Nontarget, nonmatch	47	815	8

**Social motive**			

Target, match	33	6	83

Target, nonmatch	25	4	68

Nontarget, match	656	157	10

Nontarget, nonmatch	61	806	11

**Monetary reward**			

Target, match	32	6	65

Target, nonmatch	4	25	64

Nontarget, match	678	156	10

Nontarget, nonmatch	60	806	14

**Both**			

Target, match	32	6	72

Target, nonmatch	3	26	54

Nontarget, match	706	157	10

Nontarget, nonmatch	32	806	16
